# Toxicity risk of non-target organs at risk receiving low-dose radiation: case report

**DOI:** 10.1186/1748-717X-4-71

**Published:** 2009-12-31

**Authors:** Pei-Wei Shueng, Shih-Chiang Lin, Hou-Tai Chang, Ngot-Swan Chong, Yu-Jen Chen, Li-Ying Wang, Yen-Ping Hsieh, Chen-Hsi Hsieh

**Affiliations:** 1 Department of Radiation Oncology, Far Eastern Memorial Hospital, Taipei, Taiwan; 2 Department of hematology, Far Eastern Memorial Hospital, Taipei, Taiwan; 3 Department of Chest Medicine, Division of Internal Medicine, Far Eastern Memorial Hospital, Taipei, Taiwan; 4 Institute of Traditional Medicine, School of Medicine, National Yang-Ming University, Taipei, Taiwan; 5 Department of Radiation Oncology, Mackay Memorial Hospital, Taipei, Taiwan; 6 School and Graduate Institute of Physical Therapy, College of Medicine, National Taiwan University, Taipei, Taiwan; 7 Department of Healthcare Administration, Asia University, Taichung, Taiwan; 8 Department of Radiation Oncology, National Defense Medical Center, Taiwan; 9 General Education Center, Oriental Technology Institute, Taiwan

## Abstract

The spine is the most common site for bone metastases. Radiation therapy is a common treatment for palliation of pain and for prevention or treatment of spinal cord compression. Helical tomotherapy (HT), a new image-guided intensity modulated radiotherapy (IMRT), delivers highly conformal dose distributions and provides an impressive ability to spare adjacent organs at risk, thus increasing the local control of spinal column metastases and decreasing the potential risk of critical organs under treatment. However, there are a lot of non-target organs at risk (OARs) occupied by low dose with underestimate in this modern rotational IMRT treatment. Herein, we report a case of a pathologic compression fracture of the T9 vertebra in a 55-year-old patient with cholangiocarcinoma. The patient underwent HT at a dose of 30 Gy/10 fractions delivered to T8-T10 for symptom relief. Two weeks after the radiotherapy had been completed, the first course of chemotherapy comprising gemcitabine, fluorouracil, and leucovorin was administered. After two weeks of chemotherapy, however, the patient developed progressive dyspnea. A computed tomography scan of the chest revealed an interstitial pattern with traction bronchiectasis, diffuse ground-glass opacities, and cystic change with fibrosis. Acute radiation pneumonitis was diagnosed. Oncologists should be alert to the potential risk of radiation toxicities caused by low dose off-targets and abscopal effects even with highly conformal radiotherapy.

## Background

Intensity-modulated radiotherapy (IMRT) is a powerful tool which enabled us to achieve desired dose to tumor and reducing radiation doses to critical structures simultaneously. The encouraging and safety results of patients with various sites of malignancies in the thoracic region treated by IMRT have been reported recently [1]. In addition, Gong et al. reported conventionally-fractionated image-guided intensity modulated radiotherapy (IG-IMRT) is a safe and effective treatment for cancer spinal metastasis [2].

Helical tomotherapy (HT) is a new CT-based rotational intensity modulated radiotherapy that can deliver highly conformal dose distributions with an ability to spare critical organs from radiation exposure [3]. HT is also effective and feasible for patients with multiple metastatic diseases [4].

Radiation recall phenomenon is characterized by an inflammatory reaction within the previously treated radiation field during chemotherapy treatment [5]. In humans, longer-range effects of radiotherapy occurring within or between tissues are referred to as abscopal, out-of-field, or distant bystander responses [6].

A combination of gemcitabine, 5-fluorouracil (5-FU), and leucovorin (LV) is effective in patients with unresectable or metastatic biliary tract or gallbladder adenocarcinoma [7]. Gemcitabine chemotherapy, however, can cause radiation recall followed by standard radiation therapy [8].

Herein, we present a case of radiation recall pneumonitis with simultaneous abscopal effects following highly conformal HT and gencitabine-based chemotherapy for metastatic spine lesion in a patient with metastatic cholangiocarcinoma.

## Case presentation

**Figure 1:**
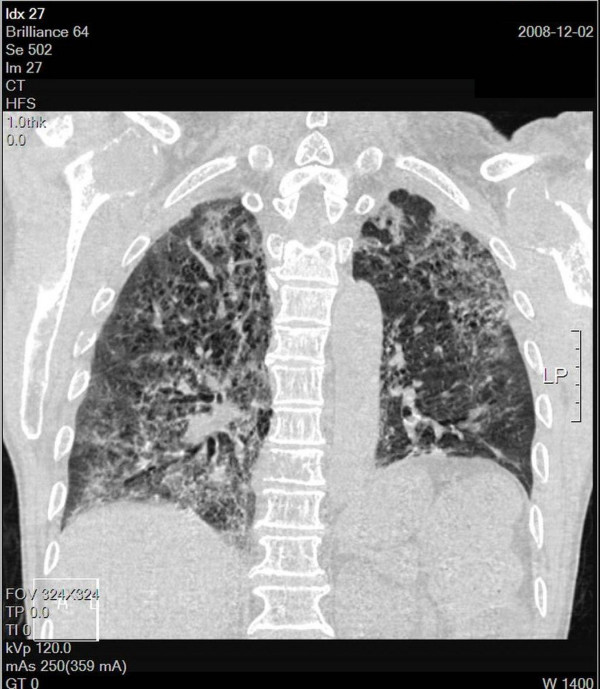
**Chest computed tomography (CT) post intubation in the MICU shows interstitial pattern with traction bronchiectasis, opacities and a diffuse ground-glass pattern, bleb formation in marginal areas, airspace consolidation and fibrosis in the bilateral lung fields**. The coronal views of chest CT.

**Figure 2:**
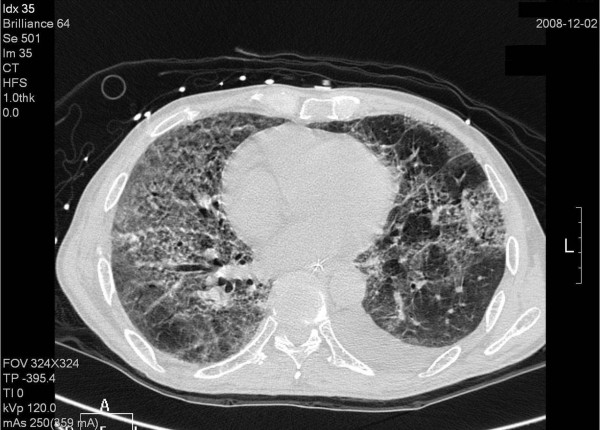
**Chest computed tomography (CT) post intubation in the MICU shows interstitial pattern with traction bronchiectasis, opacities and a diffuse ground-glass pattern, bleb formation in marginal areas, airspace consolidation and fibrosis in the bilateral lung fields**. The transverse views of chest CT.

**Figure 3:**
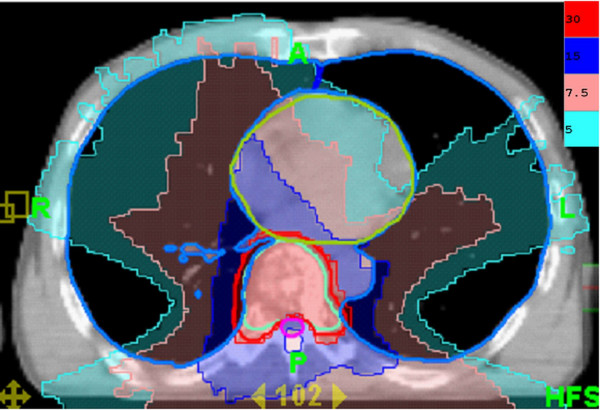
**The dose distribution of radiotherapy designed for tomotherapy**. The transverse view of low dose distribution is compatible with the recall radiation pneumonitis area.

In August, 2008, a 55-year-old man presented to the neurosurgical outpatient department of with a 2-month history of progressive claudication. The lumbar (L) -spine X-ray revealed an osteolytic lesion in the convex posterior border of the L3 vertebra. Magnetic resonance imaging (MRI) of the spine demonstrated a pathologic compression fracture with spinal canal stenosis of the thoracic (T) 9 and L3 vertebrae. Abdominal ultrasound and abdominal computed tomography (CT) scan both revealed a tumor in the left lobe of the liver. A complete blood workup showed an elevated carcinoembryonic antigen (CEA) level (33.9 ng/dl). An echo-guided biopsy of the liver tumor was performed. Histopathologic examination of the biopsy specimen showed adenocarcinoma with positive CK7 and CEA, findings compatible with primary cholangiocarcinoma. The patient underwent a left lateral sectionectomy. The pathologic diagnosis was moderately differentiated cholangiocarcinoma. Two weeks after the operation, HT with 30 Gy/10 fractions was delivered to T8-T10 for symptom relief [9]. The vertebral bodies of T8-T10 were delineated as the clinical target volume (CTV). The planning target volume (PTV) was defined as the CTV plus a 3-mm margin for tumor motion and setup uncertainty. The contoured organs at risk (OARs), dose constraints/penalty functions and planning parameters are listed in Tables 1. The field width, pitch, and modulation factor (MF) used were 2.5 cm, 0.32, and 3.0, respectively. Two weeks after the radiotherapy had been completed, the first course of gemcitabine, fluorouracil, and leucovorin was delivered. After two weeks of chemotherapy, however, the patient developed progressive dyspnea. Chest X-ray showed diffuse reticular interstitial processes in both lungs. Atypical infection was suspected. The patient was transferred to the Medical Intensive Care Unit with intubation. The blood cultures, sputum cultures, and fungus cultures were all negative. Bronchoscopy to investigate the pneumonitis was not performed at the request of the patient's family. Follow-up chest CT revealed a diffuse irregular interlobular thickness and honeycombing of both lungs (Figure 1) indicative of chronic fibrotic change. The fibrotic change in both lungs in transverse view was compatible with low dose irradiation of non-target OARs (Figure 2 and 3). Acute radiation pneumonitis was diagnosed. The following empirical antibiotics were administered: Pisutam (2.25 mg) (China Chemical & Pharmaceutical CO., LTD., Taiwan) 2 vial i.v.d. q8 h; Cravit^® ^(500 mg) (Sanofi-Aventis Deutschland GmbH, Germany) 750 mg i.v.d. qd; and Sevatrim^® ^(480 mg) (Swiss Pharmaceutical CO., LTD., Taiwan) 3 vial i.v.d. q12 h. Steroid therapy comprising methylprednisolone (40 mg), 20 mg iv. q8 h was administered for inflammatory lung disease. The patient also received antioxidants and supportive treatment simultaneously. After one month in the intensive care unit, the patient stabilized and was transferred to the hematology ward for further care.

**Table 1:** The contoured organs at risk (OARs), dose constraints/penalty functions and planning parameters of plan was listed as below

Tumor	Importance	Max Dose Constraint [Gy]	Max Dose Penalty	DVH vol [%]	DVH dose [Gy]	DVH dose [Gy]	Min Dose Penalty
**PTV_T8-T10_30Gy**	50	30.00	800	95	30.00	30.00	100

**Sensitive Structure**	**Importance**	**Max Dose Constraint [Gy]**	**Max Dose Penalty**	**DVH vol [%]**	**DVH dose [Gy]**	**DVH Point Penalty**	

**Right lung**	10	30.00	5	2	10.00	20	
**Left lung**	10	30.00	5	1	15.00	20	
**Spinal cord**	40	20.00	40	45	5.00	40	
**Heart**	10	26.00	10	20	5.00	15	
**Spleen**	5	7.00	5	10	2.00	5	
**Stomach**	1	13.00	5	10	2.00	5	
Ring*	1	30.00	50	3	28.00	20	

*Abbriviations*:

PTV = Planning target volume; Max = maximal; Min = minimal; DVH = dose-volume histogram; Vol = volume.

*The ring was a dummy structure surrounded PTV with an outer margin 2.5 cm, and a gap of 2 mm from PTV.

## Discussion

Radiation therapy (RT) is a common and safe treatment to relieve pain of symptomatic osseous metastases. In addition, RT is reserved for palliation of prevention or treatment of spinal cord compression. Generally, RT focuses on limited area for symptom relief. However, RT also is safe and effective for multiple symptomatic osseous metastatic patients as multi-fractionated wide-field radiation therapy (MF-WFRT) [10].

Radiation pneumonitis in patients undergoing treatment for lung cancer has been shown to be associated with a V20 > 20%, where V20 represents the percentage of lung volume receiving at least 20 Gy [11], and a mean lung dose > 13.6 Gy [12]. The V20 and mean lung dose in our patient were 1% and 2.7 Gy, respectively. Therefore, our plan was a safe protocol for palliative treatment of metastatic bone disease. Although the low dose around the irradiation target is usually overlooked, such as the V5 in the plan presented here (Figure 3) which was only 20%, it can potentially induce severe radiation toxicity (Figure 2).

**Figure 4:**
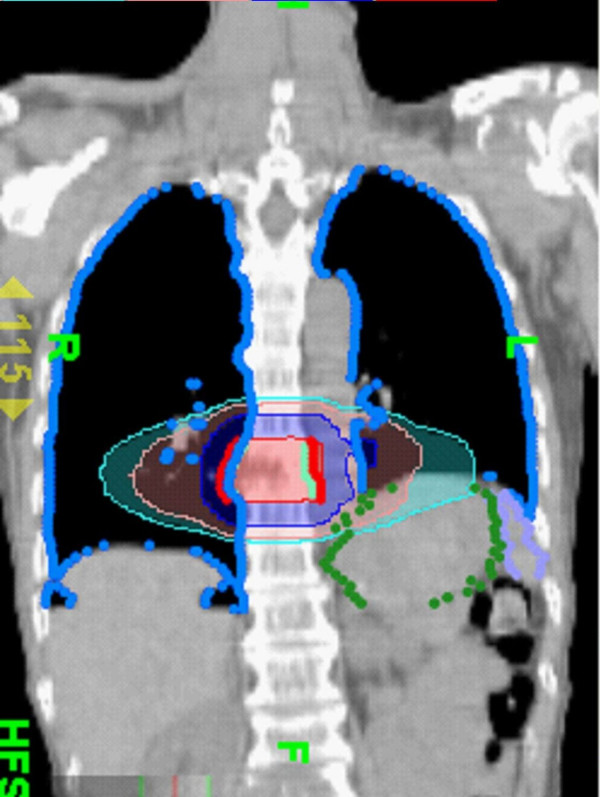
**The dose distribution of radiotherapy designed for tomotherapy**. The coronal views of dose distribution.

Although rare, gemcitabine can induce radiation recall reactions [13]. The time from gemcitabine administration to the manifestation of recall reaction ranges from 3 days to 8 months [8]. Our patient suffered from severe pulmonary toxicity 2 weeks after gemcitabine administration. Radiographic findings characteristic of radiation-induced pulmonary changes include ground-glass opacities with irregular linear opacity and interstitial thickening [14]. In our patient, the opacities with ground-glass pattern and bleb formation in the transverse views of chest CT (Figure 2) confined in the previous low dose non-target OAR field (Figure 3) indicate radiation pneumonitis recalled by gemcitabine. The diffuse irregular interlobular thickness and honeycombing of both lungs in the chest CT (Figure 1) are compatible with radiation-induced pulmonary changes, although no radiaiton was directed to these fields (Figure 4).

Khan *et al*.,[15] reported that when rat lung was partially irradiated, micronucleus formation was observed in non-irradiated areas of the lung, indicating DNA damage at these non-irradiated sites. In humans, abscopal events such as bilateral pneumonitis have been observed in humans after unilateral irradiation [16]. Additionally, a survival benefit of local control by simultaneous thoracic radiochemotherapy in the case of improved distant control due to chemotherapy and prophylactic cranial irradiation has been reported [17]. These long-range bystander responses have also been studied in a lung reconstruction model in which levels of the phosphorylated histone variant γH2AX, a marker of double-strand break (DSBs), were found to be increased, reaching a maximum by 12 to 48 h after irradiation, followed by a gradual decrease over the 7-day time course [18]. Biomolecules known to be involved in bystander responses include interleukin 6 (Il-6), Il-8, transforming growth factor-β1 (TGF-β1), and tumor necrosis factor-α (TNFα), reactive oxygen species (ROS), and reactive nitrogen species [19]. Recently, the correlation between TGF-β1 and developing radiation pneumonitis has been reported [20] and the observation could also partially response to the contribution of biomolecules on bystander responses. When distant bystander responses to radiotherapy occur during cancer treatment that the potential lung injury could be happened. If subsequent treatment is radiation recall agents that it could induce nearly fatal interstitial lung disease as the case we present here.

The low dose irradiation to non-target OARs noted in this patient is not unique to tomotherapy, rather it can occur with any technique that creates a relatively large low dose volume such as multifield IMRT, volumetric arc therapies or stereotactic radiation therapy (SRT). For example, inhomogeneity corrections have a large influence on the dose delivered to the PTV and OARs for SRT of lung tumors [21]. SRT allows for the minimization of normal tissue volume exposed to high radiation dose that is to minimize toxicity while maximizing tumor control [22]. However, even in SRT, the large amount of low dose irradiation to non-target OARs, the incidence of lung toxicity can become high has been reported by Yamashita et al. [23] Oncologists should be alert to the potential risk of low dose irradiation of non-target OARs when reviewing plans in the lung. It is important to review the low dose volumes and include the low dose volumes in the dose distribution, especially if there is a plan to give chemotherapy. Also, in cases in which there is a chance of recall within the thorax, a static field Posterior-Anterior (PA) or AP/PA, or a three-dimensional conformal radiation therapy (3DCRT) approach with fewer beams and smaller irradiated volume may be preferred for palliative (or radical treatment) to avoid this problem. In addition, even with volumetric or helical arc therapy, strong penalty functions on the lung could reduce the volume of the lung receiving even low doses.

## Conclusion

Non-target OARs can be impacted by arc therapy because of the low dose bath phenomenon. These effects can be magnified by agents known or unknown to be associated with recall effects. Optimization of planning should be considered in these situations.

## Consent

Written informed consent was obtained from the patient for publication of this case report and any accompanying images. A copy of the written consent is available for review by the Editor-in-Chief of this journal.

## Competing interests

The authors declare that they have no competing interests.

## Authors' contributions

CH Hsieh and PW Shueng carried out all CT evaluations, study design, target delineations and interpretation of the study. CH Hsieh drafted the manuscript. SC Lin and HT Chang took care of patient. NS Chong participated in data of planning preparation. YJ Chen participated in manuscript preparation. LY Wang and YP Hsieh gave advice on the work. All authors read and approved the final manuscript.
